# Supplementary material for: In-situ local phase-transitioned MoSe2 in La0.5Sr0.5CoO3-δ heterostructure and stable overall water electrolysis over 1000 hours
Source: Nat Commun. 2019 Apr 12;10:1723. doi: 10.1038/s41467-019-09339-y (PMC6461638; doi:10.1038/s41467-019-09339-y)
Supplement: Supplementary file 1 — Supplementary Information [file 41467_2019_9339_MOESM1_ESM.pdf]

Supplementary Information

In-situ Local Phase-Transitioned $MoSe_2$ in $La_{0.5}Sr_{0.5}CoO_{3-\delta}$ Heterostructure and Stable Overall Water Electrolysis over 1000 hours

Nam Khen Oh *et al*.

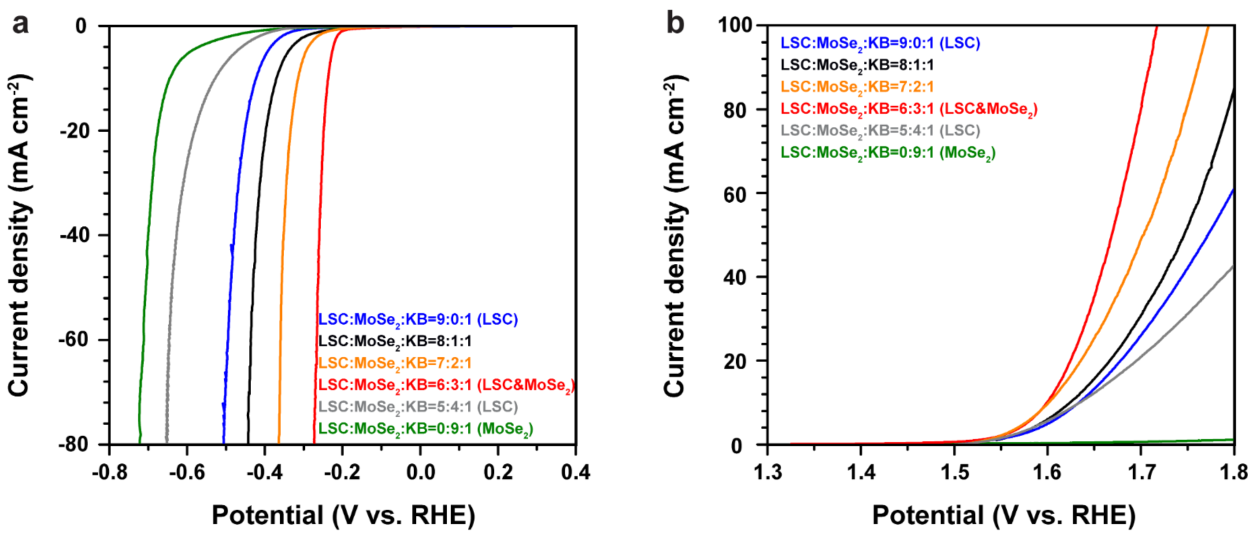

Supplementary Figure 1 HER and OER performance with various ratios of LSC and $MoSe_2$ composite catalysts. **a** HER and **b** OER polarization curves obtained by the various weight ratios of LSC and $MoSe_2$. For the preparation of catalysts, 10 wt.% of KB was included as a conductive support.

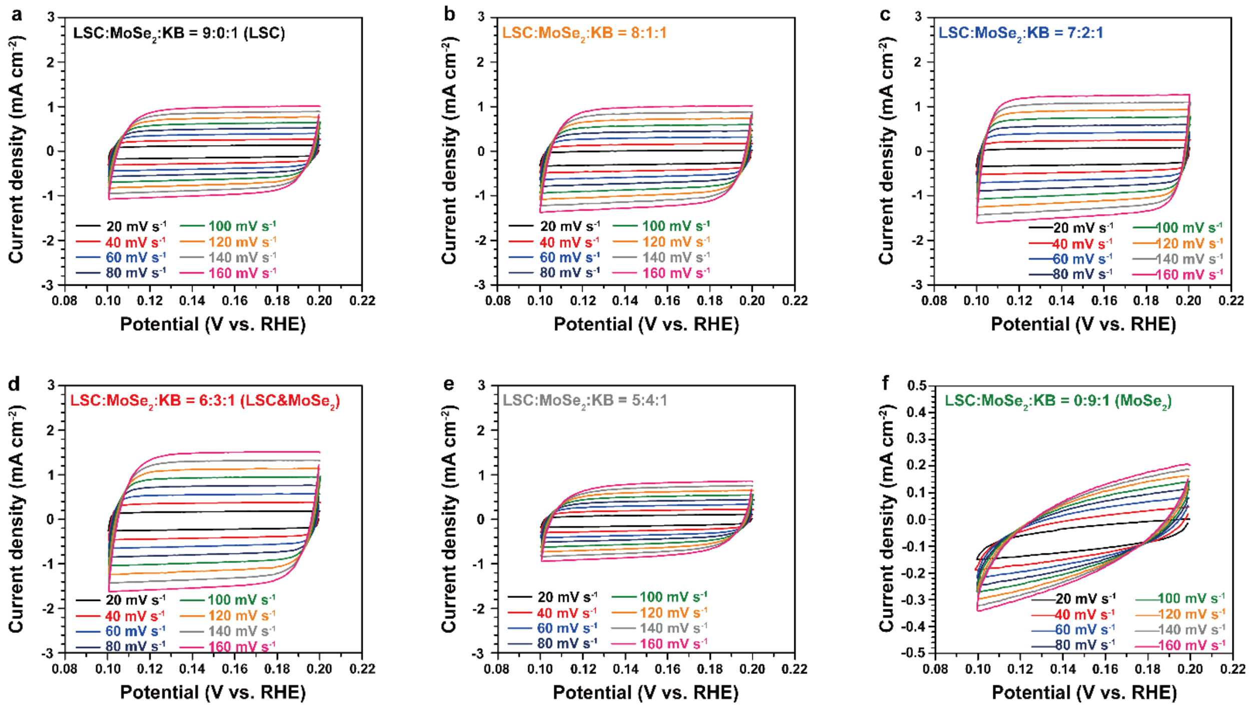

Supplementary Figure 2 Cyclic voltammograms (CVs) measured with various ratios of LSC and $MoSe_2$ composite catalysts in the double layer capacitance region at scan rates of 20, 40, 60, 80, 100, 120, 140, and 160 mV s^{-1} in 1.0 M KOH solution. The measured catalyst configurations are: **a** LSC:$MoSe_2$:KB = 9:0:1 (LSC), **b** LSC:$MoSe_2$:KB = 8:1:1, **c** LSC:$MoSe_2$:KB = 7:2:1, **d** LSC:$MoSe_2$:KB = 6:3:1 (LSC&$MoSe_2$), **e** LSC:$MoSe_2$:KB = 5:4:1, **f** LSC:$MoSe_2$:KB = 0:9:1 ($MoSe_2$).

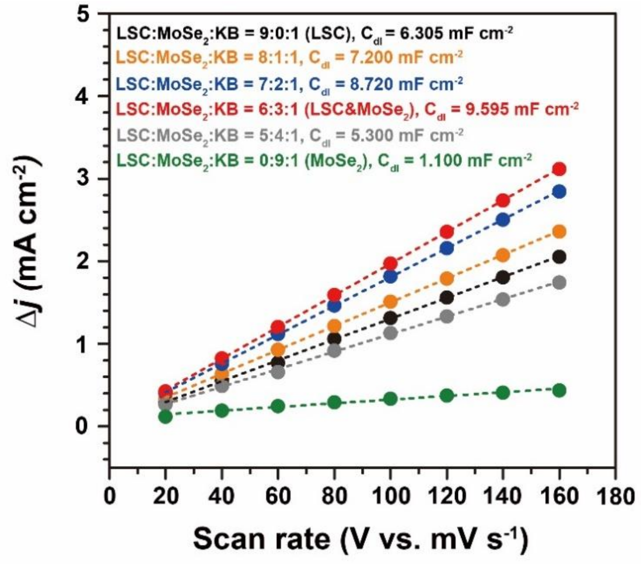

Supplementary Figure 3 Double-layer capacitance values (C_{dl}) of various LSC, $MoSe_2$, and KB configuration. Linear fitting profiles of the extraction of the C_{dl} allowing the estimation of the electrochemically active surface area ($\Delta j = (j_a\text{-}j_c)/2$).

We measured the electrochemically active surface area (ECSA) for all catalysts as shown in **Supplementary Figure 2-3**. The ECSA of LSC (LSC:$MoSe_2$:KB = 9:0:1) is measured as 6.305 mF cm^{-2} and the ECSA value increases with the addition of $MoSe_2$ upto the weight ratio 6:3:1 of LSC, $MoSe_2$, and KB (LSC&$MoSe_2$). Thus, LSC&$MoSe_2$ with the optimum ratio presents the highest ECSA value of 9.595 mF cm^{-2} as similarly observed from the HER and OER polarization profiles (**Supplementary Figure 1**). In case of $MoSe_2$ only (LSC:$MoSe_2$:KB = 0:9:1), the ECSA value is evaluated to be quite low as 1.100 mF cm^{-2} due to the electrically semiconducting properties of $MoSe_2$. For this reason, adding more $MoSe_2$ to the optimum ratio (LSC:$MoSe_2$:KB = 5:4:1) resulted in substantial reduction of the ECSA value.

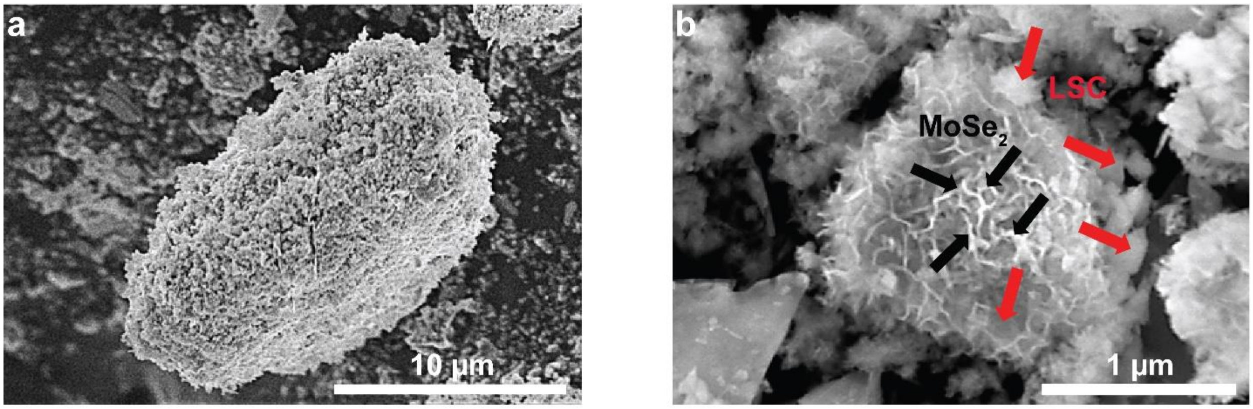

Supplementary Figure 4 SEM images of LSC&$MoSe_2$. **a** low and **b** high magnification of as-prepared LSC&$MoSe_2$.

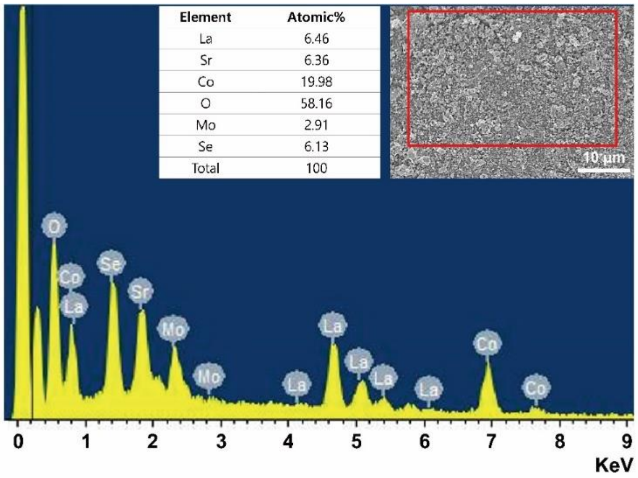

Supplementary Figure 5 SEM-EDS and elemental quantitative analysis of as-prepared LSC&$MoSe_2$. Each constituent atomic component (La, Sr, Co, O, Mo, and Se) in LSC&$MoSe_2$ is clearly observed with expected elemental ratio (The red rectangle in inset SEM image represents the selected EDS mapping area).

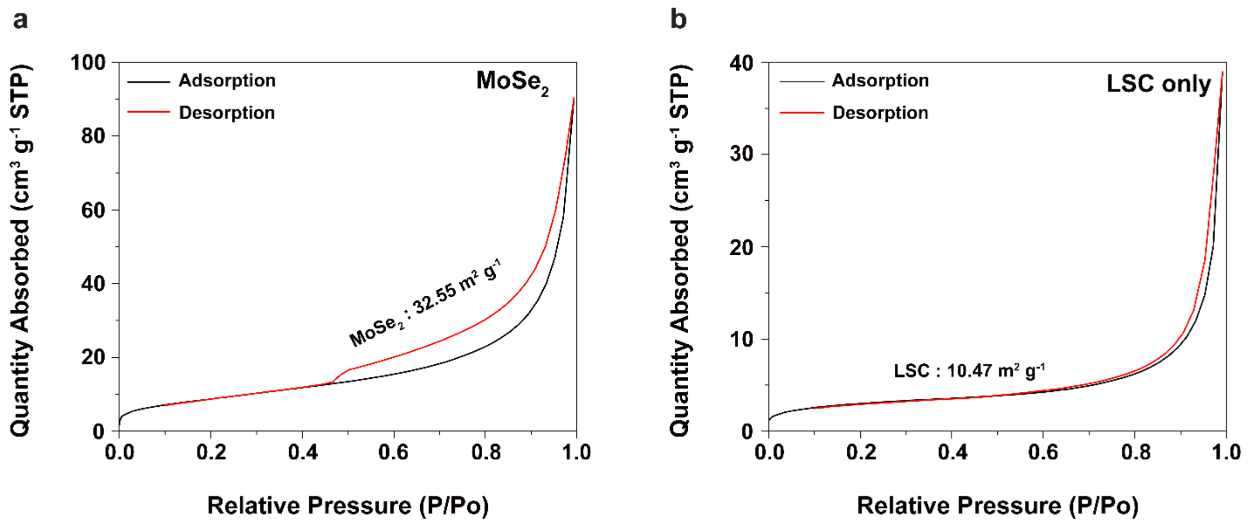

Supplementary Figure 6 BET analysis of $MoSe_2$ and LSC only. BET surface area calculated from N_2 adsorption/desorption isotherms of **a** $MoSe_2$ and **b** LSC only.

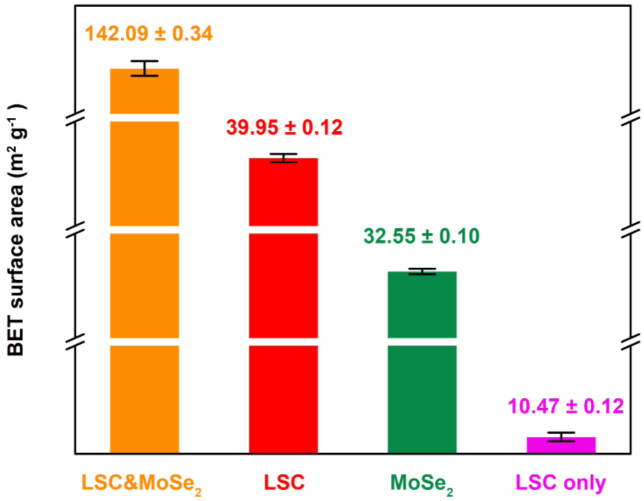

Supplementary Figure 7 Summary of BET surface area measurements of LSC&$MoSe_2$, LSC, $MoSe_2$, and LSC only. Error bars indicate the standard deviation.

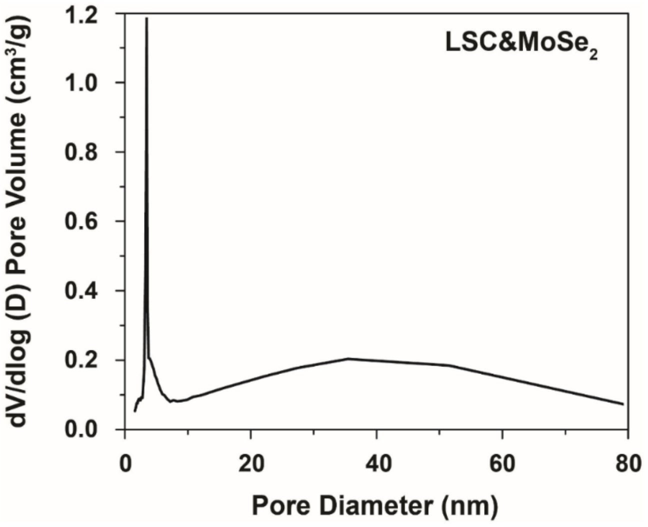

Supplementary Figure 8 BJH pore size distribution for LSC&$MoSe_2$.

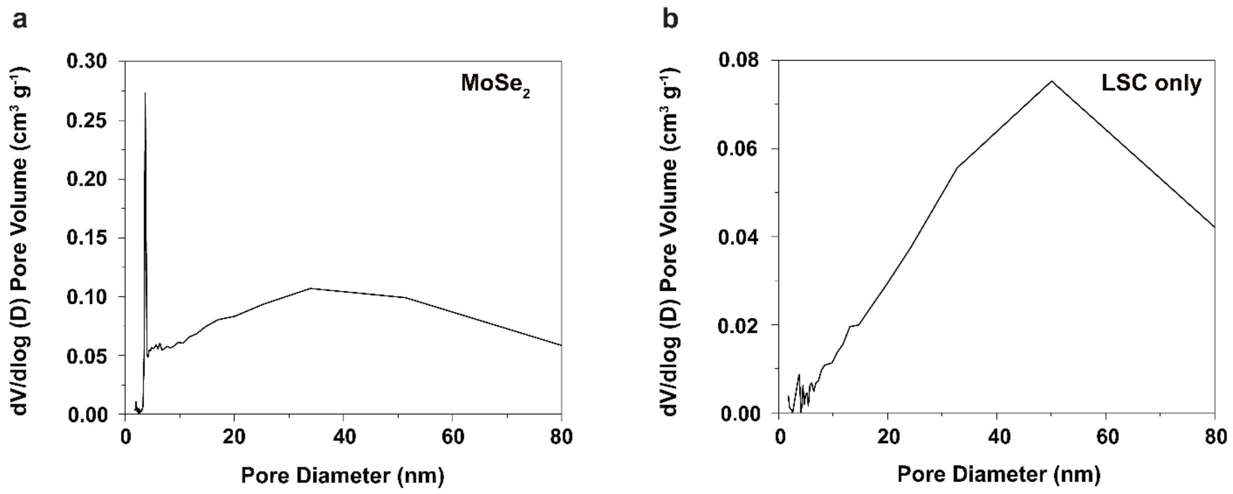

Supplementary Figure 9 BJH pore size distribution for **a** $MoSe_2$ and **b** LSC only.

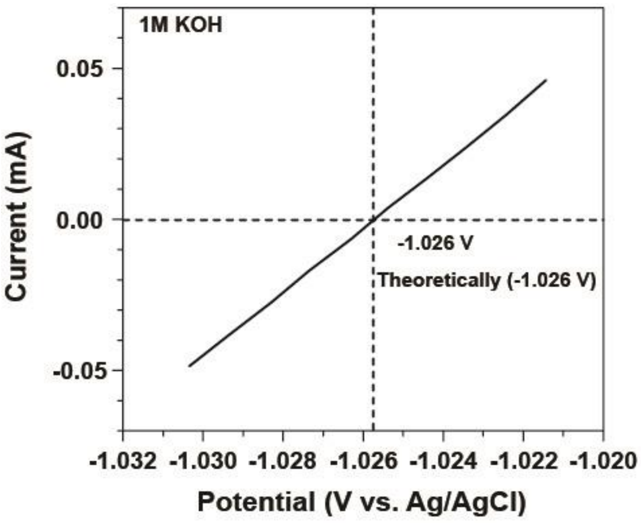

Supplementary Figure 10 Potential calibration of the Ag/AgCl reference electrode in 1 M KOH.

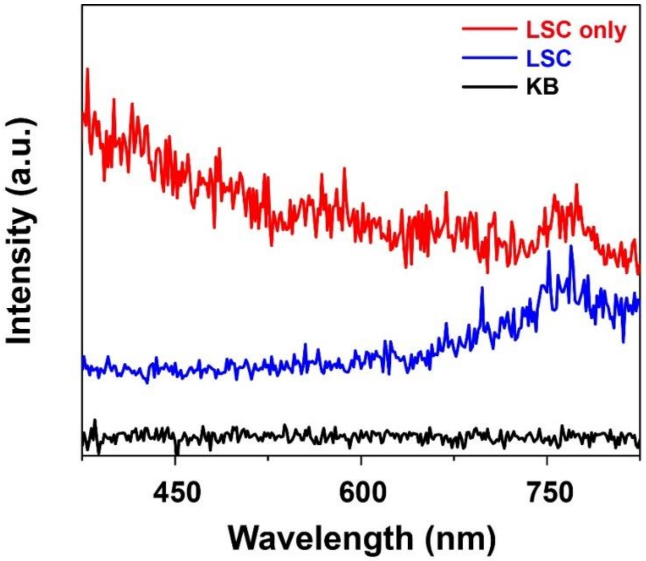

Supplementary Figure 11 Fluorescence emission spectra of LSC only, LSC, and KB.

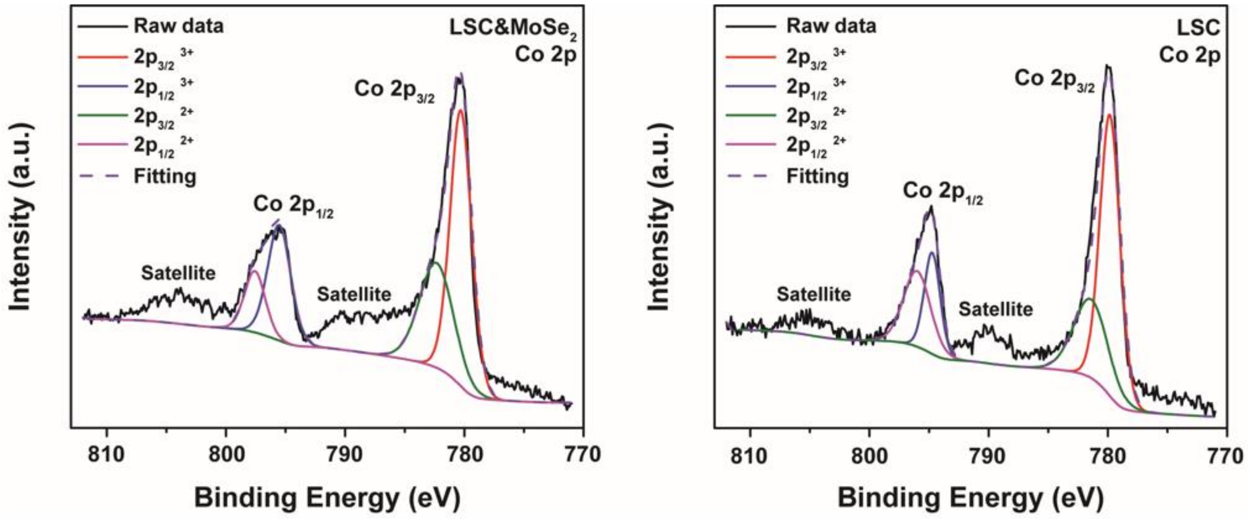

Supplementary Figure 12 X-ray photoelectron spectroscopy analysis. Co 2p XPS spectra of LSC&MoSe$_2$ and LSC consisting of two spin–orbit doublets and two satellites.

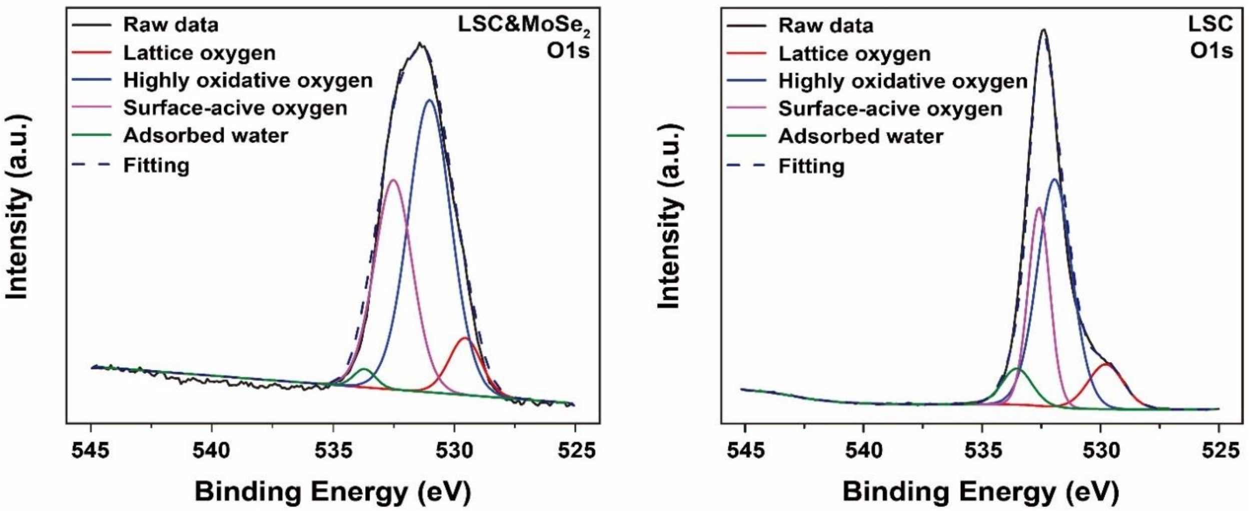

Supplementary Figure 13 X-ray photoelectron spectroscopy analysis. O 1s XPS spectra of LSC&$MoSe_2$ and LSC, which consist of lattice oxygen, highly oxidative oxygen, surface-active oxygen, and adsorbed water.

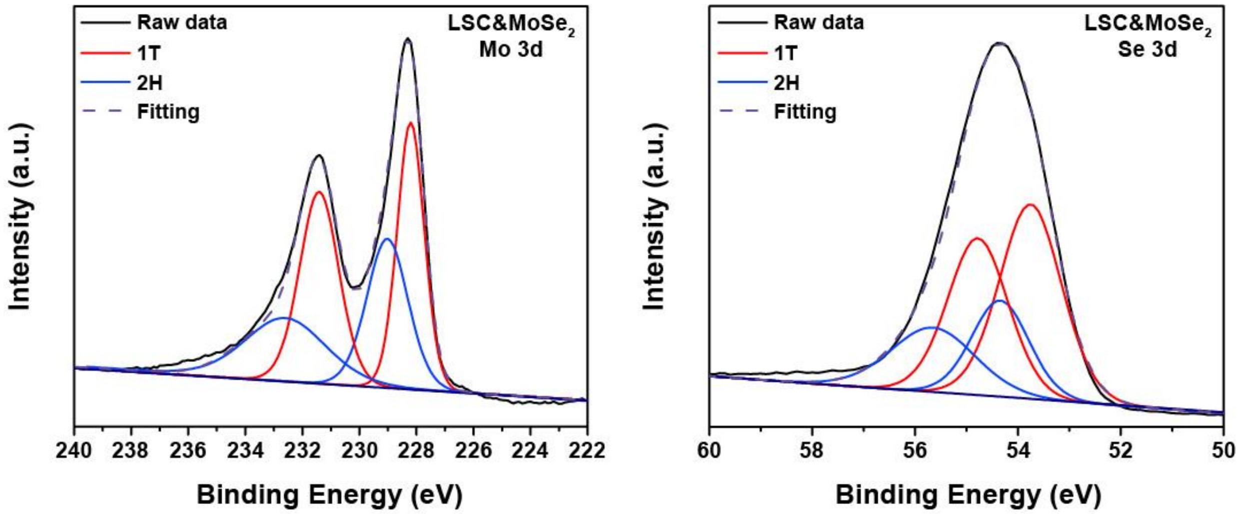

Supplementary Figure 14 X-ray photoelectron spectroscopy analysis. Mo 3d and Se 3d XPS spectra of LSC&$MoSe_2$, indicating the coexistence of 1T- and 2H-phase $MoSe_2$.

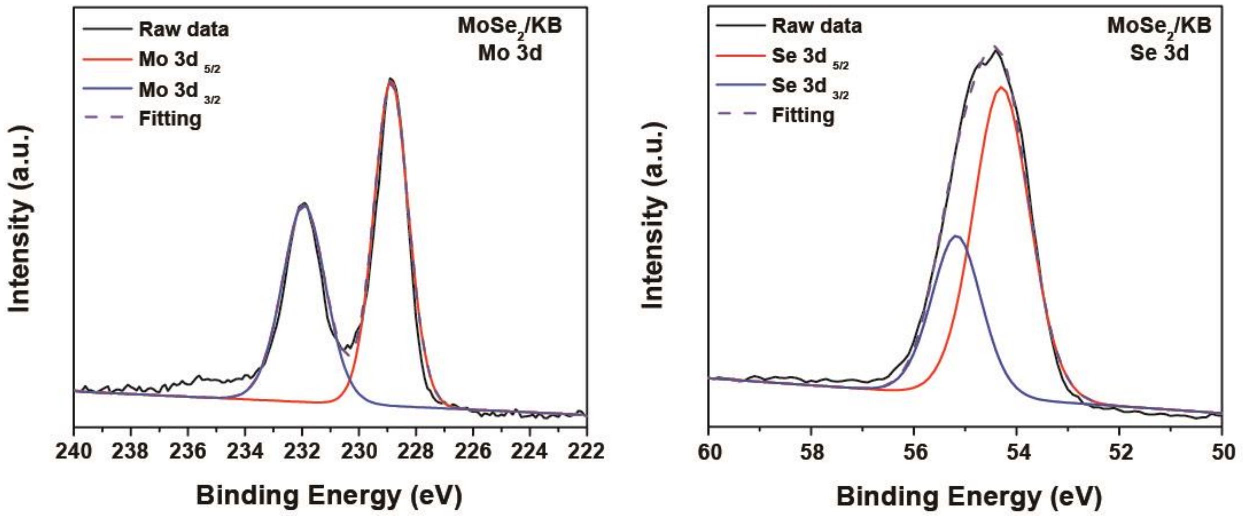

Supplementary Figure 15 X-ray photoelectron spectroscopy analysis. Mo 3d and Se 3d XPS spectra of $MoSe_2$/KB, indicating the presence of 2H-phase $MoSe_2$ only.

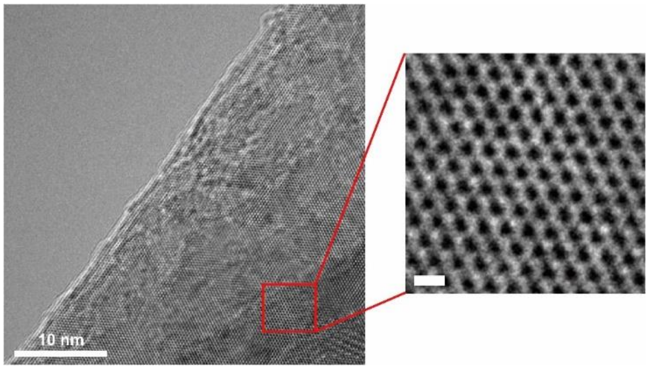

Supplementary Figure 16 HR-TEM image of $MoSe_2$/KB. The selected enlarged area shows the hexagonal crystal structure of 2H-phase $MoSe_2$ (scale bar: 0.5 nm).

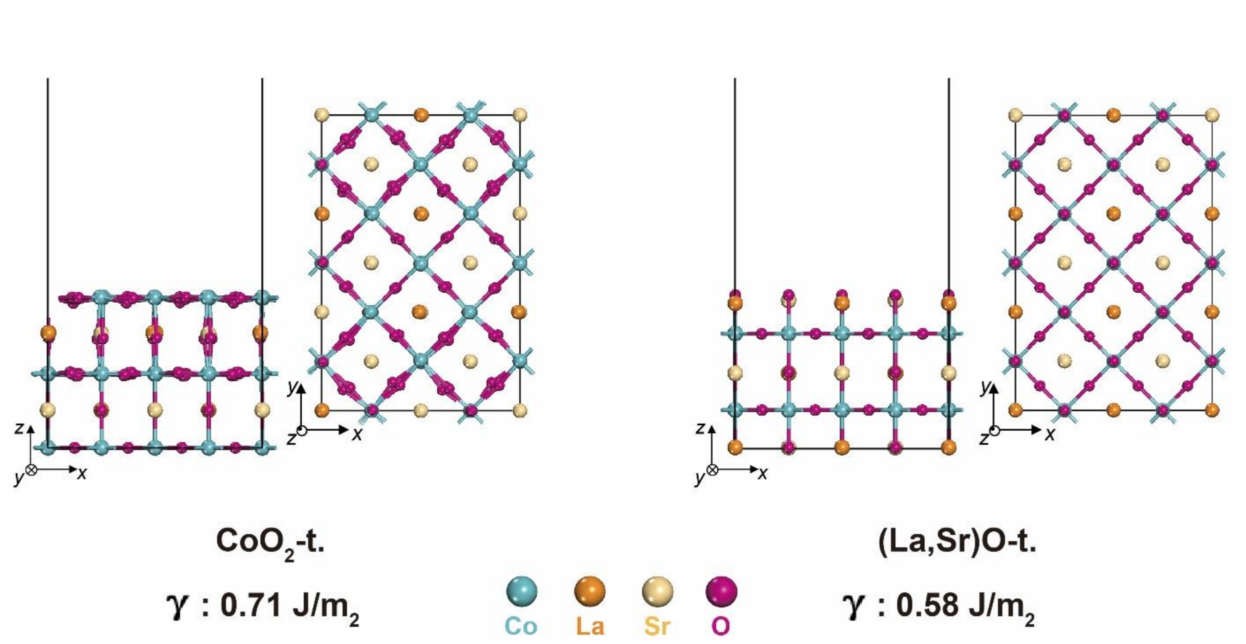

Supplementary Figure 17 Optimized structures and corresponding surface energies (γ) of symmetrical slab models for LSC (001) surface with two plausible terminations (i.e., CoO_2-t. and (La,Sr)O-t.). The red shaded box represents the fixed atoms in the two bottommost layers.

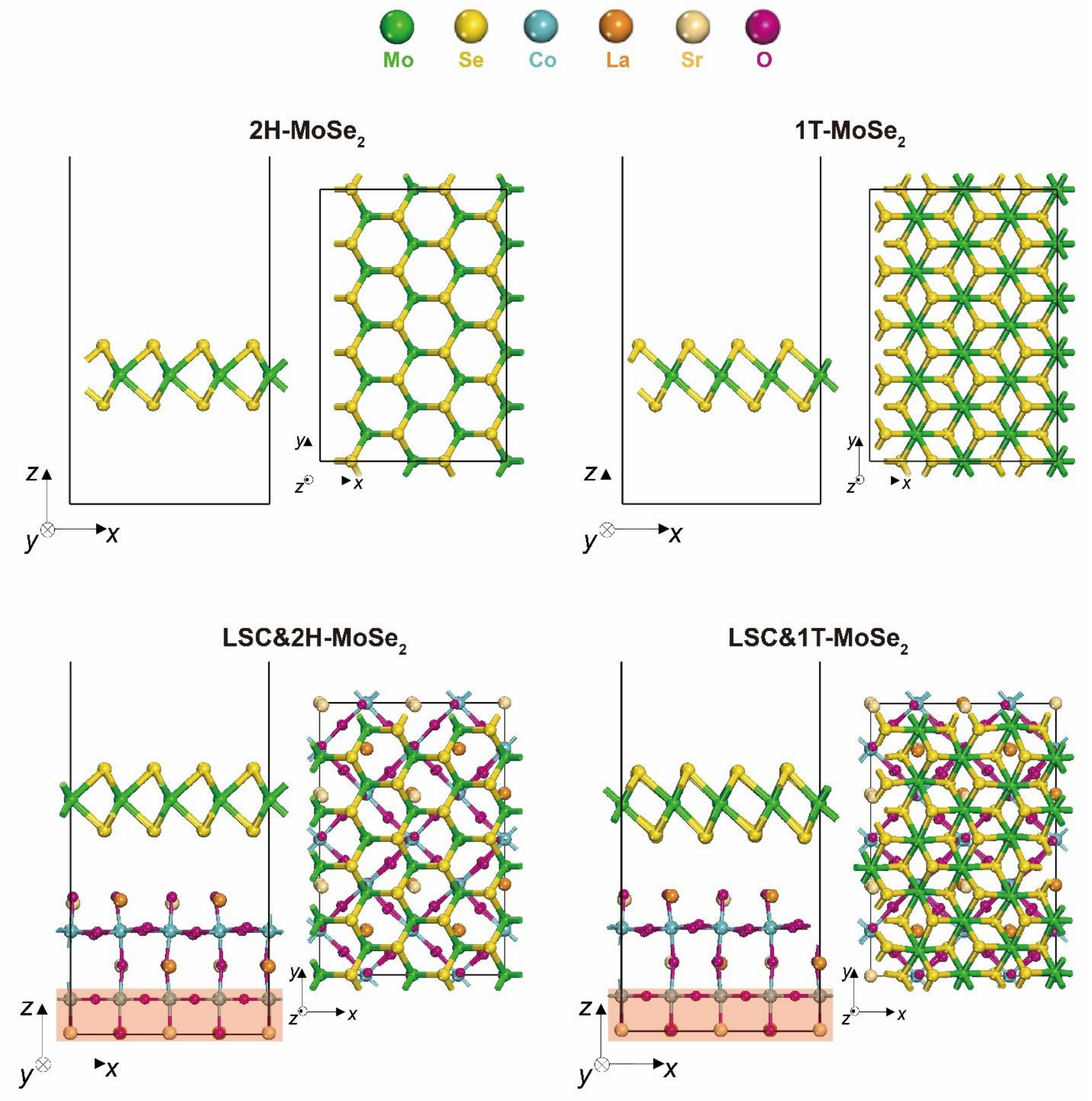

Supplementary Figure 18 Model systems used for relative energy calculation for 2H- and 1T-phase of $MoSe_2$ monolayer and LSC&$MoSe_2$ heterostructure. The red shaded box represents the fixed atoms in the two bottommost layers.

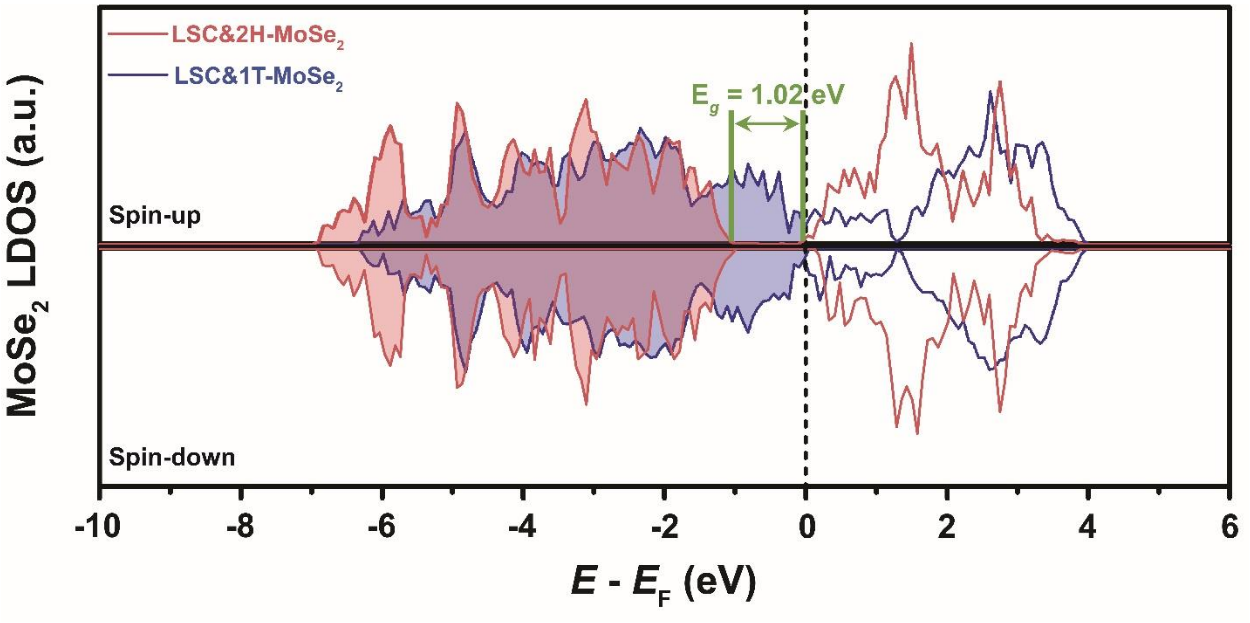

Supplementary Figure 19 Spin-up and spin-down LDOS of $MoSe_2$ in LSC&2H-$MoSe_2$ (red) and LSC&1T-$MoSe_2$ (blue) heterostructure. The shaded area represents the valence band region. The green line represents the energy gap (E_g) between valence band maximum and conduction band minimum.

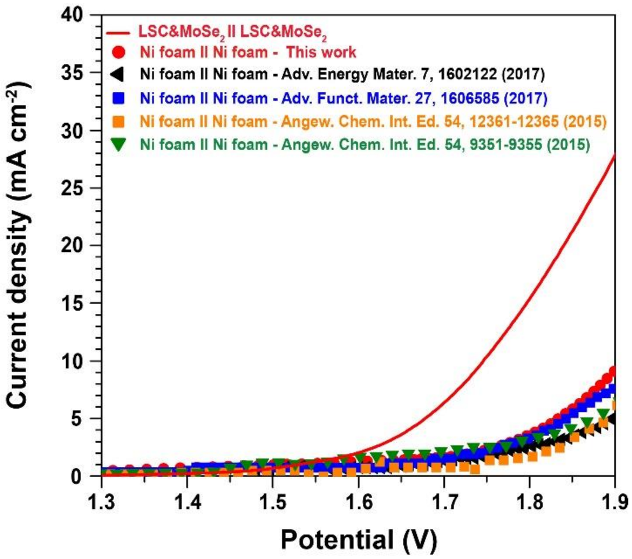

Supplementary Figure 20 Overall water electrolysis performance among various bare Ni foams compared with LSC&$MoSe_2$. Linear sweep voltammetry curves of the overall water splitting measured using the LSC&$MoSe_2$ catalyst loaded Ni foam electrode and the bare Ni foam electrode, compared with the bare Ni foam performance results reported in the literature.

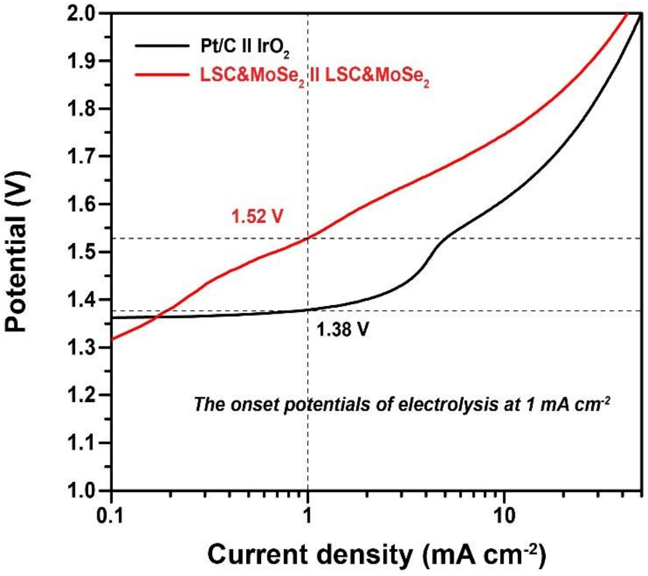

Supplementary Figure 21 Overall water splitting performance measured by Pt/C ‖ IrO_2 and $LSC\&MoSe_2$ ‖ $LSC\&MoSe_2$. Figure 6b was replotted with a logarithmic scale of current density to evaluate the onset potentials of electrolysis at a current density of 1 mA cm^{-2}.

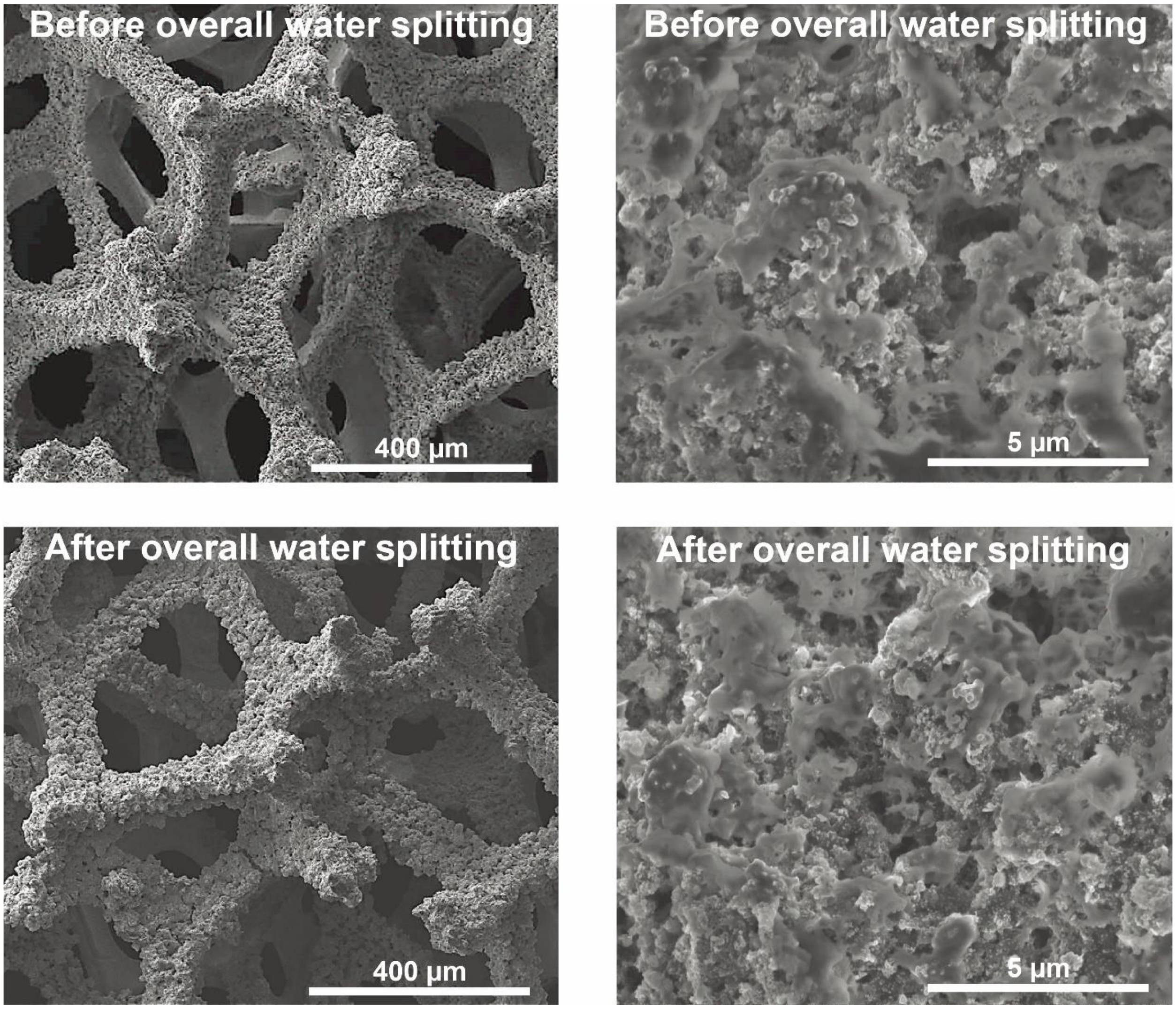

Supplementary Figure 22 SEM images of the electro-sprayed pristine LSC&$MoSe_2$ electrode and LSC&$MoSe_2$ electrode after 1,000 h of overall water splitting test. LSC&$MoSe_2$ electrode shows negligible electrode damage after the prolonged water electrolysis reaction.

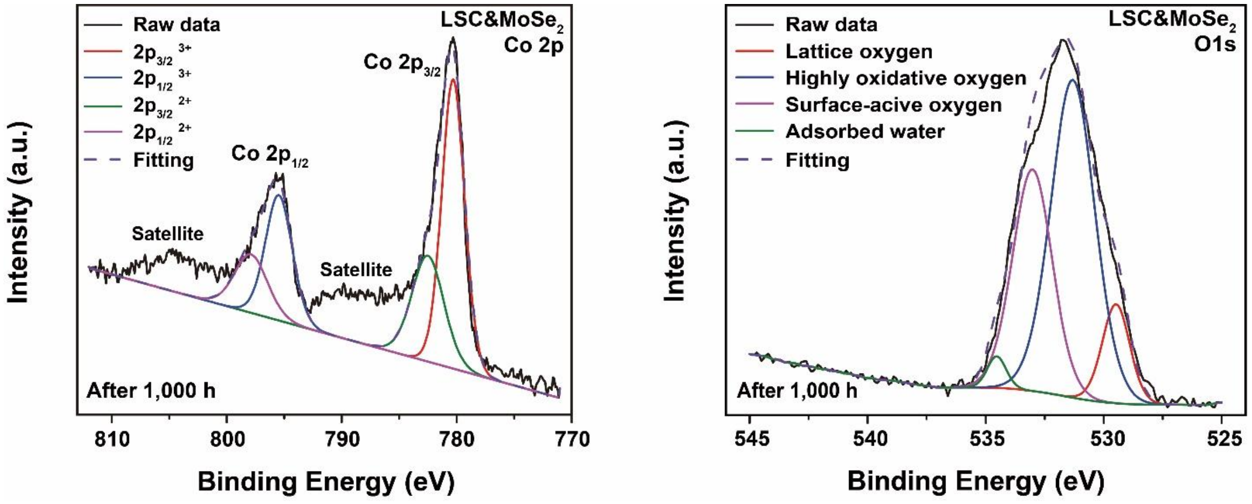

Supplementary Figure 23 X-ray photoelectron spectroscopy analysis. Co 2p and O 1s XPS spectra of LSC&$MoSe_2$ after chronopotentiometric stability test measured at 100 mA cm^{-2} for 1,000 h.

Supplementary Table 1. HER performance survey of representative electrocatalysts in 1 M KOH electrolytes.

Catalyst	Onset potential (mV)	Tafel slope (mV dec^{-1})	Electrolyte	Ref.
**This work**	**200**	**34**	**1 M KOH**	
Transition metal dichalcogenides based electrocatalyst				
$NiSe_2$ nanosheets	90	184	1 M KOH	1
MoS_{2+x} nanoparticles	200	84	1 M KOH	2
$MoS_2/MoSe_2$-0.5	180	96	1 M KOH	3
Pristine $MoSe_2$	270	135	1 M KOH	3
Pristine MoS_2	310	157	1 M KOH	3
$Ni(OH)_2/MoS_2$	210	105	1 M KOH	4
$CoSe_2/MoSe_2$	211	76	1 M KOH	5
$MoSe_2$@$Ni_{0.85}Se$	36	66	1 M KOH	6
$MoSe_2$/GCA	120	119	1 M KOH	7
ex-$MoSe_2$:$NiCl_2$	230	114	1 M KOH	8
Perovskite oxide based electrocatalyst				
$Ba_{0.5}Sr_{0.5}Co_{0.8}Fe_{0.2}O_{3-\delta}$	261	75	1M KOH	9
$Pr_{0.5}(Ba_{0.5}Sr_{0.5})_{0.5}Co_{0.8}Fe_{0.2}O_{3-\delta}$	179	45	1M KOH	9
$NdBaMn_2O_{5.5}$	200	87	1M KOH	10
$SrNb_{0.1}Co_{0.7}Fe_{0.2}O_{3-\delta}$-nanorod	210	103	1M KOH	11
$SrNb_{0.1}Co_{0.7}Fe_{0.2}O_{3-\delta}$	265	128	1M KOH	11
$Pr(Ba_{0.8}Ca_{0.2})_{0.95}(Co_{1.5}Fe_{0.5})_{0.95}Co_{0.05}O_{5+\delta}$	200	42	1M KOH	12
$La_{0.5}(Ba_{0.4}Sr_{0.4}Ca_{0.2})_{0.5}Co_{0.8}Fe_{0.2}O_{3-\delta}$	180	59	1M KOH	13
$Pr_{0.5}(Ba_{0.5}Sr_{0.5})_{0.5}Co_{0.8}Fe_{0.2}O_{3-\delta}$	180	63	1M KOH	13

Other representative non-precious metal based electrocatalyst				
Mo_2C nanoparticles	150	60	1 M KOH	14
MoB particles	150	59	1 M KOH	14
CoP nanowires/carbon cloth	38	129	1 M KOH	15
WN nanowires/carbon cloth	100	170	1 M KOH	16
NiNC–800	105	160	1 M KOH	17
FeP nanorod array	86	146	1 M KOH	18
EG/$Co_{0.85}$Se/NiFe–LDH	240	57	1 M KOH	19
CF-NG–Co	104	75	1 M KOH	20
Ni_3S_2 nanoparticle/CNTs	350	102	1 M KOH	21
Ni_2P nanoparticles	150	100	1 M KOH	22
$NiCo_2S_4$ nanowires/carbon cloth	230	141	1 M KOH	23
NiSn@C	100	145	1 M KOH	24
Carbon paper/carbon tubes/Co–S	50	131	1 M KOH	25
N, P, Co–doped graphene	350	145	1 M KOH	26
Nanostructured NiMo alloy film	10	104	1 M KOH	27

Supplementary Table 2. OER performance survey of representative electrocatalysts in 1 M KOH electrolytes.

Catalyst	Onset potential (V)	Tafel slope (mV dec^{-1})	Electrolyte	Ref.
**This work**	**1.52**	**77**	**1 M KOH**	
Transition metal dichalcogenides based electrocatalyst				
Few-layer BP	1.45	88	1 M KOH	28
MoS_2/Co_3S_4 hollow polyhedra	1.64	90.1	1 M KOH	29
Co_9S_8@MoS_2/CNFs	1.58	61	1 M KOH	30
Fe-MoS_2	1.35	126	1 M KOH	30
MoS_2 QDs	1.51	39	1 M KOH	31
MoS_2/Ni_3S_2	1.41	88	1 M KOH	32
$CoTe_2$ nanowire	1.58	67	1 M KOH	33
Perovskite oxide based electrocatalyst				
3D microporous-$LaFeO_3$	1.59	62	1 M KOH	34
3D microporous-$LaFe_{0.8}Co_{0.2}O_3$	1.57	56	1 M KOH	34
La0.7Sr0.3Co0.7Fe0.3O3-975	1.56	103	1 M KOH	35
$La_{0.7}Sr_{0.3}Co_{0.25}Mn_{0.75}O_3$-NPs-800	1.59	132	1 M KOH	36
$SrCo_{0.4}Fe_{0.2}W_{0.4}O_{3-\delta}$	1.63	58	1 M KOH	37
$SrCo_{0.4}Fe_{0.2}W_{0.05}O_{3-\delta}$	1.67	102	1 M KOH	37
Ball-milled $SrCo_{0.4}Fe_{0.2}W_{0.4}O_{3-\delta}$	1.58	81	1 M KOH	37
$La_{0.5}Sr_{0.5}Ni_{0.2}Fe_{0.8}O_{3-\delta}$	1.59	90	1 M KOH	38
$La_{0.5}Sr_{0.5}Ni_{0.4}Fe_{0.6}O_{3-\delta}$	1.47	85	1 M KOH	38
$La_{0.5}Sr_{0.5}Ni_{0.5}Fe_{0.5}O_{3-\delta}$	1.59	95	1 M KOH	38
$La_{0.5}Sr_{0.5}Ni_{0.8}Fe_{0.2}O_{3-\delta}$	1.59	96	1 M KOH	38

$Ba_{0.5}Sr_{0.5}Co_{0.8}Fe_{0.2}O_{3-\delta}$	1.55	80	1 M KOH	38
Other representative non-precious metal based electrocatalyst				
FeCo@NG/NCNT	1.54	77	1 M KOH	39
Co_3O_4–MTA	1.52	84	1 M KOH	40
NiNC–800	1.45	45	1 M KOH	17
$FeNi_3N$/Ni foam	1.43	40	1 M KOH	41
NPCN/CoNi–NCNT	1.57	165	1 M KOH	42
Ni–P	1.48	64	1 M KOH	43
Co_3O_4 NCs	1.52	101	1 M KOH	44
$Cu(OH)_2$	1.57	78.9	1 M KOH	45
MW CNT/$Cu(OH)_2$	1.65	127.9	1 M KOH	45
MW CNT/CuO–400	1.55	59.9	1 M KOH	45
FeB_2	1.48	52.4	1 M KOH	46
Ni_xB	1.54	89	1 M KOH	47
Nickel borate@Ni_3B	1.48	52	1 M KOH	48
Pristine CNTs	1.58	60	1 M KOH	49
Ni_3B	1.51	81.4	1 M KOH	50
Ni_3B–rGO	1.43	88.4	1 M KOH	50
O–CNTs	1.52	47.7	1 M KOH	51
N, O, P tri–doped porous carbon	1.52	84	1 M KOH	52

Supplementary Table 3. Summary of abbreviations for various materials studied in this work.

Abbreviation	Consisting materials
LSC only	$La_{0.5}Sr_{0.5}Co_{0.5}O_{3-\delta}$
KB	Ketjenblack carbon
$MoSe_2$	Molybdenum diselenide
LSC	$La_{0.5}Sr_{0.5}Co_{0.5}O_{3-\delta}$ + Ketjenblack carbon
LSC/$MoSe_2$	$La_{0.5}Sr_{0.5}Co_{0.5}O_{3-\delta}$ + Molybdenum diselenide
$MoSe_2$/KB	Molybdenum diselenide + Ketjenblack carbon
LSC&$MoSe_2$	$La_{0.5}Sr_{0.5}Co_{0.5}O_{3-\delta}$ + Molybdenum diselenide + Ketjenblack carbon

Supplementary Table 4. Quantitative analysis of Co^{3+}/Co^{2+} ratio in LSC&$MoSe_2$ and LSC obtained from the XPS result in Supplementary Figure 12.

	**LSC&$MoSe_2$ (atom %)**	**LSC (atom %)**
Co $2p_{3/2}$, Co^{3+}	66.2	57.8
Co $2p_{3/2}$, Co^{2+}	33.8	42.2
Co^{3+}/Co^{2+}	ca. 2.0	ca. 1.4

Supplementary Table 5. Quantitative analysis of lattice oxygen (A_o), highly oxidative oxygen (B_o), surface-active oxygen (C_o), and adsorbed water (D_o) of LSC&$MoSe_2$ and LSC obtained from the XPS result in Supplementary Figure 13.

	**LSC&$MoSe_2$ (atom %)**	**LSC (atom %)**
A_o	10.5	10.6
B_o	53.6	53.5
C_o	33.6	28.7
D_o	2.3	7.2
C_o/A_o	Ca. 3.2	Ca. 2.7

Supplementary Table 6. Quantitative analysis of 1T- and 2H-phase $MoSe_2$ in LSC&$MoSe_2$ obtained from the XPS result in Supplementary Figure 14.

**$MoSe_2$ in LSC&$MoSe_2$ (atom %)**	
1T	Ca. 58
2H	Ca. 42

Supplementary Table 7. Quantitative analysis of Co^{3+}/Co^{2+} ratio in LSC&$MoSe_2$ obtained from the XPS result in Supplementary Figure 23.

	**LSC&$MoSe_2$ (atom %)**
Co $2p_{3/2}$, Co^{3+}	65.9
Co $2p_{3/2}$, Co^{2+}	34.1
Co^{3+}/Co^{2+}	ca. 2.0

Supplementary Table 8. Quantitative analysis of lattice oxygen (A_o), highly oxidative oxygen (B_o), surface-active oxygen (C_o), and adsorbed water (D_o) of LSC&$MoSe_2$ obtained from the XPS result in Supplementary Figure 23.

	**LSC&$MoSe_2$ (atom %)**
A_o	10.5
B_o	53.6
C_o	33.6
D_o	2.3
C_o/A_o	Ca. 3.2

Supplementary Table 9. Survey of overall water splitting stability with current density and cell voltage of representative bifunctional electrocatalysts in 1 M KOH electrolytes.

Catalyst	Current density ($mA\ cm^{-2}$)	Cell Voltage (v)	Stability (h)	Electrolyte	Ref.
**This work**	**100**	**2.3**	**1000**	**1 M KOH**	
Co–P film	4	η at 0.4	25	1 M KOH	54
Ni_2P	10	1.65	10	1 M KOH	55
NiSe/NF	20	1.75	20	1 M KOH	56
$NiFeO_x$/CF	10	1.51	200	1 M KOH	57
$NiCo_2O_4$	4.5	1.65	20	1 M KOH	58
a–CoSe/Ti	10	1.7	27	1 M KOH	59
NiMo/TiM	10	1.64	10	1 M KOH	60
Ni@Cr_2O_3–NiO	20	1.5	500	1 M KOH	61
a–Co_2B	10	1.81	30	1 M KOH	62
a–Co_2B	30	2.04	10	1 M KOH	62
Nanoporous carbon/Co	20	1.57	5	1 M KOH	63
VOOH	50	1.75	50	1 M KOH	64
Ni/Mo_2C	18	1.74	10	1 M KOH	65
N–, O–, S– doped (NOSD) Co_9S_8	50	1.85	10	1 M KOH	66
$Na_{0.08}Ni_{0.9}Fe_{0.1}O_2$	16	1.6	12	1 M KOH	67
Ni@NC–800/NF	17	1.62	50	1 M KOH	17
N-Ni_3S_2/NF	20	1.55	8	1 M KOH	68
FeB_2/NF	50	1.7	4	1 M KOH	46
FeB_2/NF	10	1.55	16	1 M KOH	46
$Ni_{11}(HPO_3)_8$–$(OH)_6$	10	1.65	100	1 M KOH	69

$SrNb_{0.1}Co_{0.7}Fe_{0.2}O_{3-\delta}$	10	1.7	30	1 M KOH	11
$Cu_{0.3}Co_{2.7}P$/N doped carbon	10	η at 10 mA cm^{-2}	50	1 M KOH	70
NiFe LDH@NiCoP/NF	10	1.57	100	1 M KOH	71

Supplementary Note 1

Energy efficiency calculation

We calculated the energy efficiency of overall water electrolysis at the current density of 100 mA cm^{-2} as follows. The specific energy for producing 1 kg of hydrogen is thermodynamically given as 143 MJ kg^{-1} or 39.4 kWh kg^{-1}. Since the electrolysis cell operates near 2.3 V at 100 mA cm^{-2}, the energy required for producing 1 kg H_2 can be calculated as follows. The current density of 100 mA cm^{-2} can be expressed as 0.1 C s^{-1} cm^{-2} (∵ 1 A = 1 C s^{-1}). Then, the transferred amount of electron can be calculated as 1.036 x 10^{-6} mol s^{-1} cm^{-2} (∵ F = 96500 C mol^{-1} e^{-}), and H_2 generation rate is calculated as 5.181 x 10^{-7} mol H_2 s^{-1} cm^{-2}, which is equivalent to 3.731 x 10^{-3} g H_2 h^{-1} cm^{-2}. For producing 1 kg of H_2, the multiplication constant can be calculated as 2.681 x 10^5 h cm^2. Since the electrolysis cell operates at 0.23 W cm^{-2}, the energy required for producing 1 kg H_2 is calculated as 61.65 kWh. The energy efficiency can be calculated by dividing the theoretical specific energy for 1 kg H_2 production, *i.e.*, 39.4 kWh. Then, the energy efficiency turns out to be 63.9 %. Considering the energy efficiency for conventional alkaline electrolysis is less than 70 % with the use of platinum electrode[53], the proposed LSC&$MoSe_2$ catalyst can be considered as highly efficient for the water electrolysis.

Supplementary References

1. Liang, H. et al. Porous two–dimensional nanosheets converted from layered double hydroxides and their applications in electrocatalytic water splitting. *Chem. Mater.* **27**, 5702–5711 (2015).
2. Morales-Guio, C. G. et al. Photoelectrochemical hydrogen production in alkaline solutions using Cu_2O coated with earth–abundant hydrogen evolution catalysts. *Angew. Chem. Int. Ed.* **127**, 674–677 (2015).
3. Zhou, Q. et al. Engineering additional ddge sites on molybdenum dichalcogenides toward accelerated alkaline hydrogen evolution kinetics. *Nanoscale* **11**, 717–724 (2019).
4. Zhao, G. et al. Epitaxial growth of $Ni(OH)_2$ nanoclusters on MoS_2 nanosheets for enhanced alkaline hydrogen evolution reaction. *Nanoscale* **10**, 19074–19081 (2018).
5. Zhao, G. et al. $CoSe_2$/$MoSe_2$ Heterostructures with enriched water adsorption/dissociation sites towards enhanced alkaline hydrogen evolution reaction. *Chem. Eur. J.* **24**, 11158–11165 (2018).
6. Wang, C., Zhang, P., Lei, J., Dong, W. & Wang, J. Integrated 3D $MoSe_2$@ $Ni_{0.85}Se$ nanowire network with synergistic cooperation as highly efficient electrocatalysts for hydrogen evolution reaction in alkaline medium. *Electrochim. Acta* **246**, 712–719 (2017).
7. Huang, Y. et al. Elastic carbon aerogels reconstructed from electrospun nanofibers and graphene as three–dimensional networked matrix for efficient energy storage/conversion. *Sci. Rep.* **6**, 31541 (2016).
8. Najafi, L. et al. Doped-$MoSe_2$ nanoflakes/3d metal oxide–hydr (Oxy) oxides hybrid catalysts for pH–universal electrochemical hydrogen evolution reaction. *Adv. Energy Mater.* **8**, 1801764 (2018).

9. Xu, X. et al. A perovskite electrocatalyst for efficient hydrogen evolution reaction. *Adv. Mater.* **28**, 6442–6448 (2016).

10. Wang, J. et al. Water Splitting with an Enhanced Bifunctional Double Perovskite. *ACS catal.* **8**, 364–371 (2017).

11. Zhu, Y. et al. A perovskite nanorod as bifunctional electrocatalyst for overall water splitting. *Adv. Energy Mater.* **7**, 1602122 (2017).

12. Hua, B. et al. A coupling for success: controlled growth of Co/CoO_x nanoshoots on perovskite mesoporous nanofibres as high–performance trifunctional electrocatalysts in alkaline condition. *Nano Energy* **32**, 247–254 (2017).

13. Hua, B., Li, M., Zhang, Y. Q., Sun, Y. F., & Luo, J. L. All–in–one perovskite catalyst: smart controls of architecture and composition toward enhanced oxygen/hydrogen evolution reactions. *Adv. Energy Mater.* **7**, 1700666 (2017).

14. Vrubel, H. & Hu, X. Molybdenum boride and carbide catalyze hydrogen evolution in both acidic and basic solutions. *Angew. Chem. Int. Ed.* **124**, 12875–12878 (2012).

15. Tian, J., Liu, Q., Asiri, A. M. & Sun, X. Self-supported nanoporous cobalt phosphide nanowire arrays: an efficient 3D hydrogen–evolving cathode over the wide range of pH 0–14. *J. Am. Chem. Soc.* **136**, 7587–7590 (2014).

16. Shi, J. et al. Tungsten nitride nanorods array grown on carbon cloth as an efficient hydrogen evolution cathode at all pH values. *Electrochim. Acta* **154**, 345–351(2015).

17. Xu, Y. et al. Nickel nanoparticles encapsulated in few–layer nitrogen–doped graphene derived from metal–organic frameworks as efficient bifunctional electrocatalysts for overall water splitting. *Adv. Mater.* **29**, 1605957 (2017).

18. Liang, Y., Liu, Q., Asiri, A. M., Sun, X. & Luo, Y. Self–supported FeP nanorod arrays: a cost-effective 3D hydrogen evolution cathode with high catalytic activity. *ACS Catal*. **4**, 4065–4069 (2014).

19. Hou, Y. et al. Vertically oriented cobalt selenide/NiFe layered–double–hydroxide nanosheets supported on exfoliated graphene foil: an efficient 3D electrode for overall water splitting. *Energy Environ. Sci.* **9**, 478–483 (2016).

20. Pei, Z. et al. Construction of a hierarchical 3D Co/N–carbon electrocatalyst for efficient oxygen reduction and overall water splitting. *J. Mater. Chem. A* **6**, 489–497 (2018).

21. Lin, T. W., Liu, C. J. & Dai, C. S. Ni_3S_2/carbon nanotube nanocomposite as electrode material for hydrogen evolution reaction in alkaline electrolyte and enzyme–free glucose detection. *Appl. Catal. B: Environ.* **154**, 213–220 (2014).

22. Feng, L., Vrubel, H., Bensimon, M. & Hu, X. Easily–prepared dinickel phosphide (Ni_2P) nanoparticles as an efficient and robust electrocatalyst for hydrogen evolution. *Phys. Chem. Chem. Phys.* **16**, 5917–5921 (2014).

23. Liu, D., Lu, Q., Luo, Y., Sun, X. & Asiri, A. M. $NiCo_2S_4$ nanowires array as an efficient bifunctional electrocatalyst for full water splitting with superior activity. *Nanoscale* **7**, 15122–15126 (2015).

24. Lang, L., Shi, Y., Wang, J., Wang, F. B. & Xia, X. H. Hollow core–shell structured Ni–Sn@C nanoparticles: a novel electrocatalyst for the hydrogen evolution reaction. *ACS Appl. Mater. Interfaces* **7**, 9098–9102 (2015).

25. Zhang, X. B., Wang, J., Zhong, H. X., Wang, Z. L. & Meng, F. L. Integrated three–dimensional carbon paper/carbon tubes/cobalt–sulfide sheets as a bifunctional electrode for overall water splitting. *ACS Nano* **10**, 2342–2348 (2016).

26. Zheng, Y. et al. Toward design of synergistically active carbon–based catalysts for electrocatalytic hydrogen evolution. *ACS Nano* **8**, 5290–5296 (2014).

27. Xu, W., Lu, Z., Wan, P., Kuang, Y. & Sun, X. High-performance water electrolysis system with double nanostructured superaerophobic electrodes. *Small* **12**, 2492–2498 (2016).

28. Ren, X. et al. Few-layer black phosphorus nanosheets as electrocatalysts for highly efficient oxygen evolution reaction. *Adv. Energy Mater.* **7**, 1700396 (2017).

29. Lei, X., Yu, K., Li, H. & Zhu, Z. A functional design and synthesization for electrocatalytic hydrogen evolution material on MoS_2/Co_3S_4 hybrid hollow nanostructure. *Electrochim. Acta*, **269**, 262–273 (2018).

30. Tang, B. et al. Simultaneous edge and electronic control of MoS_2 nanosheets through Fe doping for an efficient oxygen evolution reaction. *Nanoscale* **10**, 20113–20119 (2018).

31. Mohanty, B. et al. MoS_2 quantum dots as efficient catalyst materials for the oxygen evolution reaction. *ACS Catal.* **8**, 1683–1689 (2018).

32. Zhang, J. et al. Interface engineering of MoS_2/Ni_3S_2 heterostructures for highly enhanced electrochemical overall–water–splitting activity. *Angew. Chem. Int. Ed.* **128**, 6814–6819 (2016).

33. Ji, L. et al. Hierarchical $CoTe_2$ nanowire array: an effective oxygen evolution catalyst in alkaline media. *ACS Sustainable Chem. Eng.* **6**, 4481–4485 (2018).

34. Dai, J. et al. Enabling high and stable electrocatalytic activity of iron-based perovskite oxides for water splitting by combined bulk doping and morphology designing. *Adv. Mater. Interfaces* **6**, 1801317 (2018).

35. Majee, R., Chakraborty, S., Salunke, H. G. & Bhattacharyya, S. Maneuvering the physical properties and spin states to enhance the activity of La–Sr–Co–Fe–O perovskite oxide

nanoparticles in electrochemical water oxidation. *ACS Appl. Energy Mater.* **1**, 3342–3350 (2018).

36. Wan, M. et al. Building block nanoparticles engineering induces multi–element perovskite hollow nanofibers structure evolution to trigger enhanced oxygen evolution. *Electrochim. Acta* **279**, 301–310 (2018).
37. Chen, G. et al. Ultrahigh–performance tungsten–doped perovskites for the oxygen evolution reaction. *J. Mater. Chem. A* **6**, 9854–9859 (2018).
38. Wang, C. C., Cheng, Y., Ianni, E., Jiang, S. P. & Lin, B. A highly active and stable $La_{0.5}Sr_{0.5}Ni_{0.4}Fe_{0.6}O_{3-\delta}$ perovskite electrocatalyst for oxygen evolution reaction in alkaline media. *Electrochim. Acta* **246**, 997–1003 (2017).
39. Du, B., Meng, Q. T., Sha, J. Q. & Li, J. S. Facile synthesis of FeCo alloys encapsulated in nitrogen–doped graphite/carbon nanotube hybrids: efficient bi–functional electrocatalysts for oxygen and hydrogen evolution reactions. *New J. Chem.* **42**, 3409–3414 (2018).
40. Zhu, Y. P., Ma, T. Y., Jaroniec, M. & Qiao, S. Z. Self–templating synthesis of hollow Co_3O_4 microtube arrays for highly efficient water electrolysis. *Angew. Chem. Int. Ed.* **56**, 1324–1328 (2017).
41. Zhang, B. et al. Iron–nickel nitride nanostructures in situ grown on surface–redox–etching nickel foam: Efficient and ultrasustainable electrocatalysts for overall water splitting. *Chem. Mater.* **28**, 6934–6941 (2016).
42. Hou, Y. et al. Strongly coupled 3D hybrids of N–doped porous carbon nanosheet/CoNi alloy–encapsulated carbon nanotubes for enhanced electrocatalysis. *Small* **11**, 5940–5948 (2015).

43. Yu, X. Y., Feng, Y., Guan, B., Lou, X. W. D. & Paik, U. Carbon coated porous nickel phosphides nanoplates for highly efficient oxygen evolution reaction. *Energy Environ. Sci.* **9**, 1246–1250 (2016).

44. Du, S. et al. Co_3O_4 nanocrystal ink printed on carbon fiber paper as a large–area electrode for electrochemical water splitting. *Chem. Commun.* **51**, 8066–8069 (2015).

45. Chinnappan, A., Ji, D., Baskar, C., Qin, X. & Ramakrishna, S. 3–Dimensional MWCNT/CuO nanostructures use as an electrochemical catalyst for oxygen evolution reaction. *J. Alloys Compd*. **735**, 2311–2317 (2018).

46. Li, H. et al. Earth–abundant iron diboride (FeB_2) nanoparticles as highly active bifunctional electrocatalysts for overall water splitting. *Adv. Energy Mater.* **7**, 1700513 (2017).

47. Masa, J. et al. Ultrathin high surface area nickel boride (Ni_xB) nanosheets as highly efficient electrocatalyst for oxygen evolution. *Adv. Energy Mater.* **7**, 1700381 (2017).

48. Jiang, J. et al. Highly active and durable electrocatalytic water oxidation by a $NiB_{0.45}/NiO_x$ core–shell heterostructured nanoparticulate film. *Nano Energy* **38**, 175–184 (2017).

49. Cheng, Y et al. Pristine carbon nanotubes as non–metal electrocatalysts for oxygen evolution reaction of water splitting. *Appl. Catal. B: Environ.* **163**, 96–104 (2015).

50. Arivu, M., Masud, J., Umapathi, S. & Nath, M. Facile synthesis of Ni_3B/rGO nanocomposite as an efficient electrocatalyst for the oxygen evolution reaction in alkaline media. *Electrochem. Commun.* **86**, 121–125 (2018).

51. Li, L. et al. Unraveling oxygen evolution reaction on carbon–based electrocatalysts: effect of oxygen doping on adsorption of oxygenated intermediates. *ACS Energy Lett.* **2**, 294–300 (2017).

52. Lai, J. et al. Unprecedented metal–free 3D porous carbonaceous electrodes for full water splitting. *Energy Environ. Sci.* **9**, 1210–1214 (2016).

53. Stolten, D. & Emonts, B. *Hydrogen Science and Engineering: Materials, Process, Systems, and Technology* (John Wiley & Sons, 2016).

54. Jiang, N., You, B., Sheng, M. & Sun, Y. Electrodeposited cobalt-phosphorous–derived films as competent bifunctional catalysts for overall water splitting. *Angew. Chem. Int. Ed.* **127**, 6349–6352 (2015).

55. Stern, L. A., Feng, L., Song, F. & Hu, X. Ni_2P as a Janus catalyst for water splitting: the oxygen evolution activity of Ni_2P nanoparticles. *Energy Environ. Sci.* **8**, 2347–2351 (2015).

56. Tang, C., Cheng, N., Pu, Z., Xing, W. & Sun, X. NiSe nanowire film supported on nickel foam: an efficient and stable 3D bifunctional electrode for full water splitting. *Angew. Chem. Int. Ed.* **127**, 9483–9487 (2015).

57. Wang, H. et al. Bifunctional non–noble metal oxide nanoparticle electrocatalysts through lithium–induced conversion for overall water splitting. *Nat. Commun.* **6**, 7261 (2015).

58. Gao, X. et al. Hierarchical $NiCo_2O_4$ hollow microcuboids as bifunctional electrocatalysts for overall water–splitting. *Angew. Chem. Int. Ed.* **128**, 6398–6402 (2016).

59. Liu, T., Liu, Q., Asiri, A. M., Luo, Y. & Sun, X. An amorphous CoSe film behaves as an active and stable full water–splitting electrocatalyst under strongly alkaline conditions. *Chem. Commun.* **51**, 16683–16686 (2015).

60. Tian, J. et al. Self–supported NiMo hollow nanorod array: an efficient 3D bifunctional catalytic electrode for overall water splitting. *J. Mater. Chem. A* **3**, 20056–20059 (2015).

61. Gong, M. et al. Blending Cr_2O_3 into a NiO–Ni electrocatalyst for sustained water splitting. *Angew. Chem. Int. Ed.* **54**, 11989–11993 (2015).

62. Masa, J. et al. Amorphous cobalt boride (Co_2B) as a highly efficient nonprecious catalyst for electrochemical water splitting: oxygen and hydrogen evolution. *Adv. Energy Mater.* **6**, 1502313 (2016).

63. Wang, H. et al. Synthesis of single–crystal–like nanoporous carbon membranes and their application in overall water splitting. *Nat. Commun.* **8**, 13592 (2017).

64. Shi, H., Liang, H., Ming, F. & Wang, Z. Efficient overall water–splitting electrocatalysis using lepidocrocite VOOH hollow nanospheres. *Angew. Chem. Int. Ed.* **129**, 588–592 (2017).

65. Yu, Z. Y. et al. A one–dimensional porous carbon–supported Ni/Mo_2C dual catalyst for efficient water splitting. *Chem. Sci.* **8**, 968–973 (2017).

66. Huang, S. et al. N–, O–, and S–tridoped carbon–encapsulated Co_9S_8 nanomaterials: efficient bifunctional electrocatalysts for overall water splitting. *Adv. Funct. Mater.* **27**, 1606585 (2017).

67. Weng, B. et al. A layered $Na_{1-x}Ni_yFe_{1-y}O_2$ double oxide oxygen evolution reaction electrocatalyst for highly efficient water–splitting. *Energy Environ. Sci.* **10**, 121–128 (2017).

68. Chen, P. et al. 3D nitrogen–anion–decorated nickel sulfides for highly efficient overall water splitting. *Adv. Mater.* **29**, 1701584 (2017).

69. Menezes, P. W. et al. A structurally versatile nickel phosphite acting as a robust bifunctional electrocatalyst for overall water splitting. *Energy Environ. Sci.* **11**, 1287–1298 (2018).

70. Song, J. et al. Bimetallic cobalt–based phosphide zeolitic imidazolate framework: CoP_x phase–dependent electrical conductivity and hydrogen atom adsorption energy for efficient overall water splitting. *Adv. Energy Mater.* **7**, 1601555 (2017).

71. Zhang, H. et al. Bifunctional heterostructure assembly of NiFe LDH nanosheets on NiCoP nanowires for highly efficient and stable overall water splitting. *Adv. Funct. Mater.* **28**, 1706847 (2018).
