# Supplementary material for: In-situ local phase-transitioned MoSe2 in La0.5Sr0.5CoO3-δ heterostructure and stable overall water electrolysis over 1000 hours
Source: Nat Commun. 2019 Apr 12;10:1723. doi: 10.1038/s41467-019-09339-y (PMC6461638; doi:10.1038/s41467-019-09339-y)
Supplement: Supplementary file 3 — Description of Additional Supplementary Information [file 41467_2019_9339_MOESM3_ESM.pdf]

Description of Additional Supplementary Files

File Name: Supplementary Movie 1
Description: Overall water electrolysis test of LSC&$MoSe_2$ || LSC&$MoSe_2$
